# A novel mechanism causing imbalance of mitochondrial fusion and fission in human myopathies

**DOI:** 10.1093/hmg/ddy033

**Published:** 2018-01-19

**Authors:** Marina Bartsakoulia, Angela Pyle, Diego Troncoso-Chandía, Josefa Vial-Brizzi, Marysol V Paz-Fiblas, Jennifer Duff, Helen Griffin, Veronika Boczonadi, Hanns Lochmüller, Stephanie Kleinle, Patrick F Chinnery, Sarah Grünert, Janbernd Kirschner, Verónica Eisner, Rita Horvath

**Affiliations:** 1 Wellcome Centre for Mitochondrial Research, Institute of Genetic Medicine, Newcastle University, Newcastle upon Tyne NE1 3BZ, UK; 2 Department of Cellular and Molecular Biology, School of Biological Sciences, Pontificia Universidad Católica de Chile, Santiago, Chile; 3 Medical Genetics Center, Munich, Germany; 4 MRC Mitochondrial Biology Unit & Department of Clinical Neurosciences, University of Cambridge, Cambridge Biomedical Campus, Cambridge CB2 0QQ, UK; 5 Department of General Pediatrics, Adolescent Medicine and Neonatology, Medical Center – University of Freiburg, Freiburg, Germany; 6 Department of Neuropediatrics and Muscle Disorders, Faculty of Medicine, Medical Center – University of Freiburg, Freiburg, Germany

## Abstract

Mitochondrial dynamics play an important role in cellular homeostasis and a variety of human diseases are linked to its dysregulated function. Here, we describe a 15-year-old boy with a novel disease caused by altered mitochondrial dynamics. The patient was the second child of consanguineous Jewish parents. He developed progressive muscle weakness and exercise intolerance at 6 years of age. His muscle biopsy revealed mitochondrial myopathy with numerous ragged red and cytochrome *c* oxidase (COX) negative fibers and combined respiratory chain complex I and IV deficiency. MtDNA copy number was elevated and no deletions of the mtDNA were detected in muscle DNA. Whole exome sequencing identified a homozygous nonsense mutation (p.Q92*) in the *MIEF2* gene encoding the mitochondrial dynamics protein of 49 kDa (MID49). Immunoblotting revealed increased levels of proteins promoting mitochondrial fusion (MFN2, OPA1) and decreased levels of the fission protein DRP1. Fibroblasts of the patient showed elongated mitochondria, and significantly higher frequency of fusion events, mtDNA abundance and aberrant mitochondrial cristae ultrastructure, compared with controls. Thus, our data suggest that mutations in *MIEF2* result in imbalanced mitochondrial dynamics and a combined respiratory chain enzyme defect in skeletal muscle, leading to mitochondrial myopathy.

## Introduction

Mitochondria are essential organelles for maintaining cellular energy production in the form of ATP through oxidative phosphorylation (OXPHOS) and they play a role in the fundamental physiological cellular processes such as energy balance, modulation of calcium signaling, cellular redox and the normal function of many significant biosynthetic pathways (1). Mitochondria are constantly re-shaping organelles that move along cytoskeletal tracks, undergoing fusion and fission events to build interconnected networks, assisting the cell to adapt to its ever-changing physiological condition (2).

In skeletal muscle, mitochondria play key roles in metabolic regulation, fiber type specificity and intracellular calcium homeostasis (3). Diverse mitochondrial pathologies have been linked to skeletal muscle dysfunction and mutations in mitochondrial dynamics proteins lead to variable mitochondrial diseases affecting the nervous system and skeletal muscle. Patients carrying mutations affecting the inner mitochondrial membrane fusion protein, OPA1, associated with Autosomal Dominant Optical Atrophy (ADOA), are characterized by early-onset blindness and develop late-onset myopathy (4) and altered muscle mitochondrial ATP synthesis (5). In addition, mutations affecting the outer mitochondrial membrane (OMM) fusion protein, MFN2, are associated with autosomal dominant axonal Charcot-Marie-Tooth Type 2 (CMT2) neuropathy (6–7). Patients carrying *MFN2* mutations associated with CMT2 also develop late onset myopathy (8), suggesting that altered mitochondrial dynamics compromises the organelle’s function especially in neuronal cells and in skeletal muscle.

Mitochondrial fission requires the recruitment of a large GTPase to the OMM from the cytosol, called dynamin-related protein 1 (DRP1). DRP1 exists as dimer or tetramer in the cytosol and when it binds to the OMM assembles into higher-ordered complexes requiring GTP hydrolysis (9–11), and this directs its constriction and scission (12–13).

In mammals, three adaptors (MFF, MID49 and MID51) recruit DRP1 to the OMM. Recruitment of DRP1 to the OMM is significantly reduced in MFF-null cells, indicating that MFF is essential to mitochondrial fission (14–15). Recent studies have suggested that MID49 and MID51, which are encoded by the genes *MIEF2* (*SMCR7*) and *MIEF1* (*SMCR7L*), respectively, act as receptors of DRP1. The genes encoding MID49 and MID51 are paralogues to each other sharing 45% of sequence identity (16) and differentially expressed in tissues during different developmental stages (17). Both proteins are anchored in the OMM and can recruit DRP1 independently to MFF and FIS1 (16,18). Finally, MID49 and MID51 are specific mitochondrial adaptors of DRP1 in contrast to MFF and FIS1 which are also present in peroxisomes (18).

Here we report a patient with mitochondrial myopathy due to a homozygous mutation in MID49 (*MIEF2, SMCR7*) with combined respiratory chain enzyme defect and augmented mitochondrial fusion in the patient`s skeletal muscle and fibroblasts.

## Results

### Clinical presentation

The 15-year-old boy is the second child of consanguineous Jewish parents. The older sister is healthy. At the age of 6 years he first presented with progressive muscle weakness, intermittent muscle pain and exercise intolerance. Creatine kinase (CK) activity in serum was elevated (1283 U/l, normal < 170 U/l). At that time, a brain MRI was performed with unremarkable results, and dystrophinopathy was excluded genetically by MLPA. Pompe’s disease was excluded by enzymatic testing of alpha glucosidase activity in dried blood spots. Within the following years he received regular physiotherapy, but his muscular symptoms aggravated. At the age of 10 years he first presented to our centre for further diagnostics. He was able to walk about 200 meters and to climb about 10 steps without a break. Ophthalmological examination was repeatedly normal, EMG has not been performed because the reflexes were normal and there was no clinical sign of neuropathy.

Clinical investigation revealed a proximal muscle weakness without any signs of other organ involvement. The CK level was again elevated (760 U/l, normal < 217 U/l). Cardiologic evaluation was normal. A muscle biopsy was performed and revealed a mitochondrial myopathy. A metabolic screening including organic acids in urine and acylcarnitines in dried blood spots was performed, but showed a normal pattern and did not indicate multiple acyl-CoA dehydrogenase deficiency (MADD). The patient was started on riboflavin (100 mg/day p. o.) and coenzyme Q10 (120 mg/day). Physiotherapeutic treatment was continued once per week. Under this regimen both muscle pain as well as muscle strength improved (walking range 800 meters, 2–3 stairs, cycling 12 kilometers). During follow up CK levels varied between 377 and 1996 U/l (normal < 247 U/l).

### Muscle biopsy analysis

**Figure 1.**
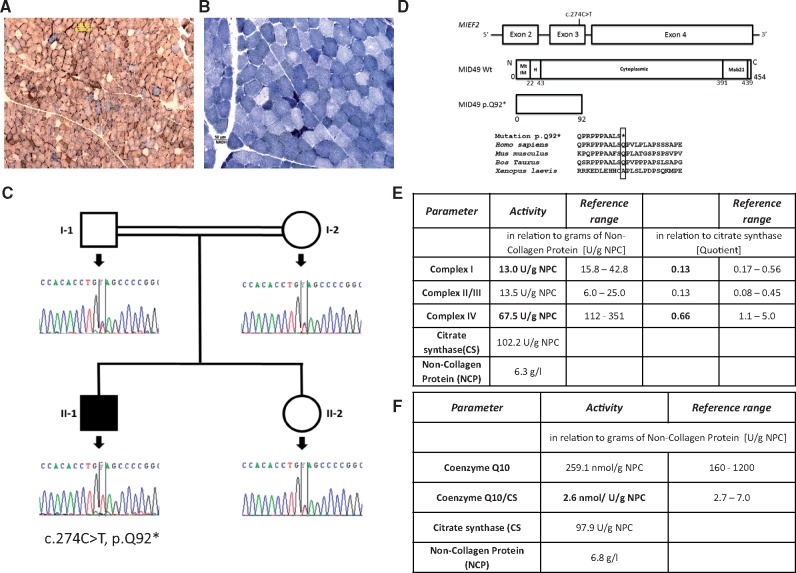
Diagnostic testing of respiratory chain function in skeletal muscle and genetic studies. (**A**) Simultaneous staining of muscle fibres for cytochrome oxidase (COX) and succinate dehydrogenase (SDH), (**B**) Nicotinamide adenine dinucleotide (NADH) staining of muscle fibres. Dark blue muscle fibres represent increased mitochondrial proliferation, pale muscle fibres illustrate deficient muscle fibres. (**C**) Pedigree diagram and Sanger sequencing results showing the segregation of the *MIEF2* c.274C>T, p.Q92* mutation. (**D**) Schematic showing *MIEF2* gene structure, MID49 wildtype protein structure [domains represented as follows: Mitochondrial Intermembrane (MtIM), Helical (H), Cytoplasmic and Mab21] and MID49 mutant p.Q92* structure including conservational analyses of the amino acid among species. (**E**) Biochemical measurement of respiratory chain enzymes. (**F**) Coenzyme Q10 in the muscle biopsy of the patient. Abnormal values are marked in bold.

Muscle biopsy revealed mitochondrial myopathy with numerous ragged red fibres (data not-shown) and COX negative fibres (Fig. 1A). In addition, the muscle fibres showed heterogeneous staining intensity of NADH dehydrogenase indicative of aberrant mitochondrial function in certain fibres (low blue) and elevated mitochondrial mass in small, likely regenerative fibres (dark blue) (Fig. 1B). Biochemical measurement showed deficiencies of multiple respiratory chain enzymes (Fig. 1E and F).

### Molecular genetic studies

Diagnostic testing excluded mutations and deletions of the mtDNA and no depletion was detected, rather mtDNA copy number was three times higher than the control in skeletal muscle. WES identified a homozygous stop mutation in the *MIEF2* gene (NM_148886) (c.274C > T, p.Q92*), encoding the mitochondrial elongation factor 2, MID49*.* Sanger sequencing was undertaken to validate the variants and confirm that these segregated with the disease in the family (Fig. 1C). This mutation is predicted to be pathogenic, because it results in a premature stop leading to loss of the MID49 protein (Fig. 1D). The variant is very rare and is not found in ExAC or gnomAD. The patient also carries a homozygous likely benign (class 2 after the classification of the American College of Medical Genetics and Genomics) *ETFA* mutation (c.20C > T, p.P7L). It has been seen in ExAc in 9 out of 15 286 cases in heterozygous state. Mutations in *ETFA* cause multiple acyl-CoA dehydrogenase deficiency (MADD), however metabolic screening in the patient revealed no MADD-typical pattern and lipid accumulation was also absent in muscle excluding this diagnosis.

Quantitative PCR for detecting mRNA of *MIEF2* and *MIEF1* in the patient`s fibroblasts showed severe reduction of *MIEF2* mRNA, suggesting nonsense-mediated decay, while *MIEF1* (MID51) was not significantly altered (Fig. 3D).

### Biochemical respiratory chain enzyme analysis and immunoblotting in skeletal muscle

**Figure 2.**
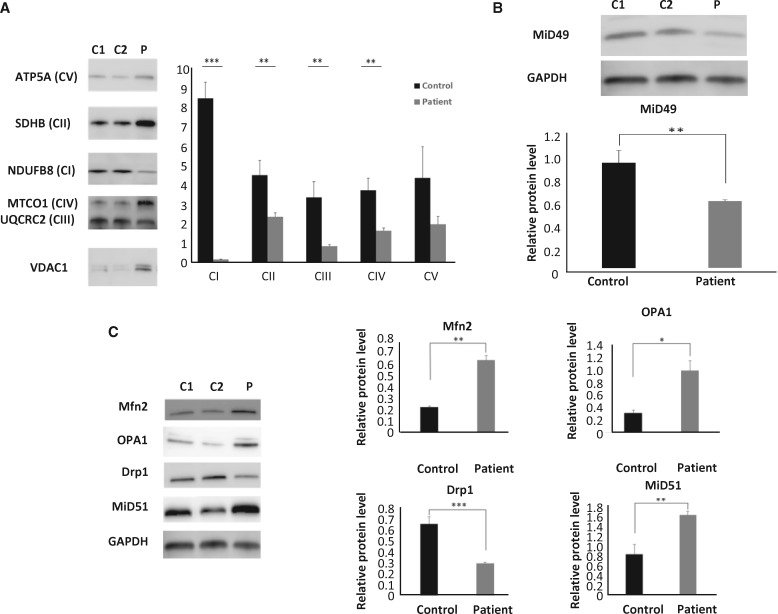
Mitochondrial function in skeletal muscle. (**A**) Immunoblot analysis of OXPHOS complex subunits performed on total protein lysate from skeletal muscle of the patient (p.Q92*) and controls (*n*=3). (**B**) Immunoblotting detected significantly decreased expression of MID49 protein in the patient`s skeletal muscle when compared with controls (*n=*3). (**C**) Quantification of MFN2, OPA1, DRP1 and MID51 protein levels from total protein lysates extracted from muscle biopsies of the patient and controls (*n=*3).

Biochemical measurement showed deficiencies of multiple respiratory chain enzymes (Fig. 1E). Subunits of complexes I, II, III and IV were significantly decreased in the patient’s skeletal muscle, once normalized by mitochondrial mass content that showed elevation, estimated by VDAC1 relative levels. Complex V protein levels did not change significantly (Fig. 2A).

MID49 protein in the muscle biopsy of the patient showed significantly decreased levels, when compared with controls (Fig. 2B). Based on our sequencing studies, a total absence of the MID49 band was expected. As MID49 and MID51 share 45% homology, it is possible that the polyclonal antibody is detecting some MID51 in this patient’s lysate.

Given the relevance of MID49 in the maintenance of mitochondrial dynamics, we evaluated the protein levels of MFN2, OPA1, DRP1 and MID51 in total protein lysates extracted from the patient’s and controls’ skeletal muscle. These data revealed significant increases in the relative levels of MFN2 (*P =* 0.002, unpaired *t*-test) and OPA1 (*P =* 0.02, unpaired *t*-test). DRP1 levels were significantly decreased (*P =* 0.0005, unpaired *t*-test) in the patient’s skeletal muscle, while MID51 was significantly increased in the same tissue (Fig. 2C). Thus, these data suggest that both fusion and fission proteins adapt in the patient to compensate the absence of MID49.

### Mitochondrial functional studies in fibroblasts

**Figure 3.**
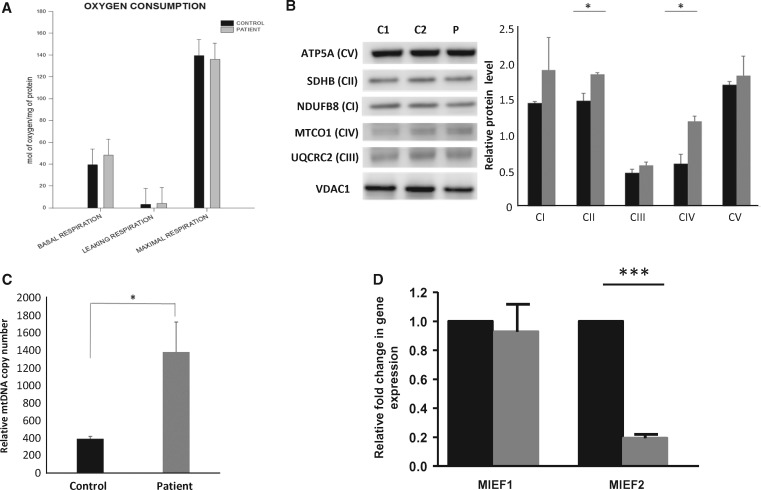
Mitochondrial functional studies in patient fibroblasts. (**A**) Oxygen consumption in fibroblasts carrying the homozygous nonsense mutation c.274C>T, p.Q92* in *MIEF2* (MID49). Black and grey bars represent the mean values of control and patient’s primary fibroblast cells respectively. The corrected oxygen consumption by the non-mitochondrial respiration (NMR) is represented as basal respiration, leaking respiration and maximal respiration. These data are representative of three different biological replicates. (**B**) Immunoblot analysis of OXPHOS complex subunits performed on total protein lysate of fibroblasts from the patient and controls (*n=*3). (**C**) Quantification of the relative mtDNA copy number shows a significant increase in the patient’s fibroblasts when compared with controls. (*n=*3)**.** (**D**) Quantitative PCR of *MIEF1* and *MIEF2* mRNA in control (black bars) and patient (grey bars) fibroblasts (*n=*3). The graphs represent relative fold change in gene expression to housekeeping genes *GAPDH* and *β-Actin*.

To investigate functional effects of the *MIEF2* (MID49) variant, primary fibroblasts from the patient and control subjects were analysed. Oxygen consumption of the patient fibroblasts (representative of three biological replicates) showed a slight increase in basal respiration rate (22% increase) compared with the control fibroblasts whilst the levels of maximal respiration were slightly decreased (3% decrease) compared with controls (Fig. 3A). These results were not statistically significant. Elevated protein levels were detected in all OXPHOS complexes (Fig. 3B), as measured by immunoblotting. A significant increase was observed in the relative protein levels of CII and CIV (*P =* 0.02, *P =* 0.016 respectively, unpaired *t*-test).

Relative mtDNA copy number was measured in DNA extracted from the patient's fibroblasts and controls. MtDNA copy number was significantly increased (*P =* 0.01, unpaired *t*-test) in the patient when compared with control (Fig. 3C).

To further demonstrate the depletion of MID49 we performed immunoblotting, detecting significantly decreased protein expression levels (*P =* 0.01, unpaired *t*-test) of MID49 in the patient’s fibroblasts when compared with two control cell lines (Supplementary Material, Fig. S1A). These results are representative of three biological replicates. Immunoblotting for enriched mitochondrial fraction from the patient and control fibroblasts also showed depletion of the MID49 protein (data not shown).

We measured the proteins involved in mitochondrial fusion and fission, including MFN2, OPA1, DRP1 and MID51 in whole protein lysate isolated from the patient’s fibroblasts and compared with controls. The patient showed a slightly decreased level of DRP1 and MID51 protein compared with control fibroblasts. A slight increase in expression of MFN2 and OPA1 was observed in the patient, when compared with controls, but this was not significant. The results are representative of three biological replicates (Supplementary Material, Fig. S1B).

### *In vivo* mitochondrial continuity and fusion analysis

**Figure 4.**
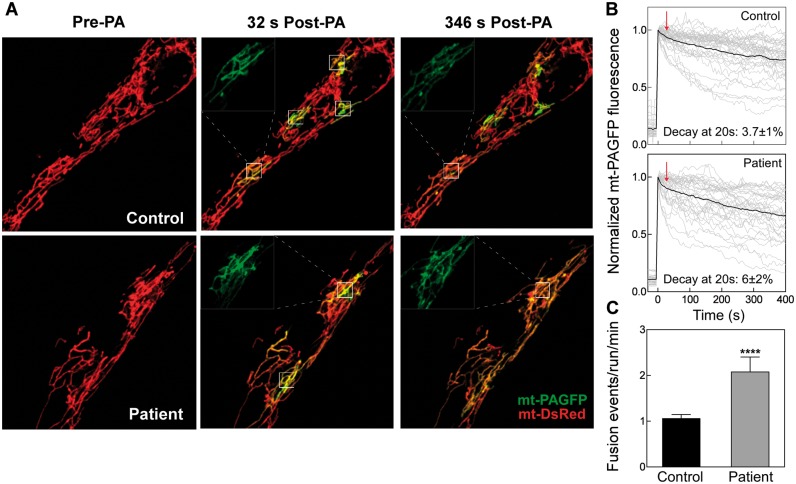
Mitochondrial continuity and fusion are elevated in human mutant fibroblasts (MID49 p.Q92*). (**A**) Cells were transfected with mtDsRed and mtPA-GFP codifying plasmids and images by confocal microscopy. Representative cells before and after photoactivation of 5x5 regions interest (ROIs: white squares). The images display the continuity among mitochondria evidenced by the diffusion of photoconverted PA-GFP towards neighbouring mitochondrial out of ROIs. (**B**) mtPA-GFP fluorescence decay evaluated inside the photoactivation area. Grey curves, individual regions; black curves represent the mean. (**C**) Frequency of fusion events. *n=* 29 Control and 24 Patient cells. Data from at least four independent experiments.

Next, we evaluated the dynamics of mitochondria, testing continuity and fusion frequency. Fibroblasts of the patient with the *MID49* mutations and controls were transiently transfected to express mitochondrial matrix-targeting fluorescent proteins mtDsRed and photoswitchable protein mtPA-GFP, and tested by *in vivo* confocal microscopy. Mitochondrial morphology of MID49 p.Q92* cells showed elongated pattern (Fig. 4A, left panel), consistent with a decrease in fission protein MID49. In addition, we tested mitochondrial continuity and fusion events frequency by photoactivation of mtPA-GFP within confined regions of interest (ROI, 25 μm^2^), and followed the diffusion of the photoswitched protein, towards the vicinity of the ROI (Fig. 4B). As expected, mitochondrial continuity, measured as the decay of mtPA-GFP fluorescence intensity in the ROIs, augmented in the MID49 p.Q92* carrying cells, and else, the fusion events frequency was significantly elevated in these cells. Thus, the morphology and connectivity of mitochondria are increased in MID49 p.Q92* carrying cells, likely consistent with an impaired fission phenotype. In addition, fusion of mitochondria is also induced, suggesting a compensatory effect to keep mitochondrial quality control.

To further confirm that the hyperfused mitochondrial pattern is caused by the absence of MID49 and not by an unrelated adaptive response, we rescued MID49 by exogenous expression of human MID49 (hMID49-GFP) in the patient’s fibroblasts (Supplementary Material, Fig. S2A). Our data show that hMID49-GFP shifted the hyperfused mitochondrial phenotype observed in the patient, to intermediate and semi-connected mitochondria population, comparable to mitochondrial morphology in control cells (Supplementary Material, Fig. S2B). In addition, we performed fluorescence recovery after photobleaching (FRAP) experiments to study mitochondrial continuity, which is known to be supported by mitochondrial fusion and motility. We found that rescue of hMID49 in the patient’s cells showed a significant decrease in the FRAP kinetics, again comparable to control cells (Supplementary Material, Fig. S2C and D). Expression of hMID49-GFP did not alter mitochondrial morphology or continuity in control human fibroblasts. Thus, our findings show that mitochondrial dynamics defects presented in MID49 p.Q92* carrying fibroblasts are a consequence of the absence of the protein and not of unrelated causes, confirming the pathogenicity of the mutation.

### Ultrastructure studies

**Figure 5.**
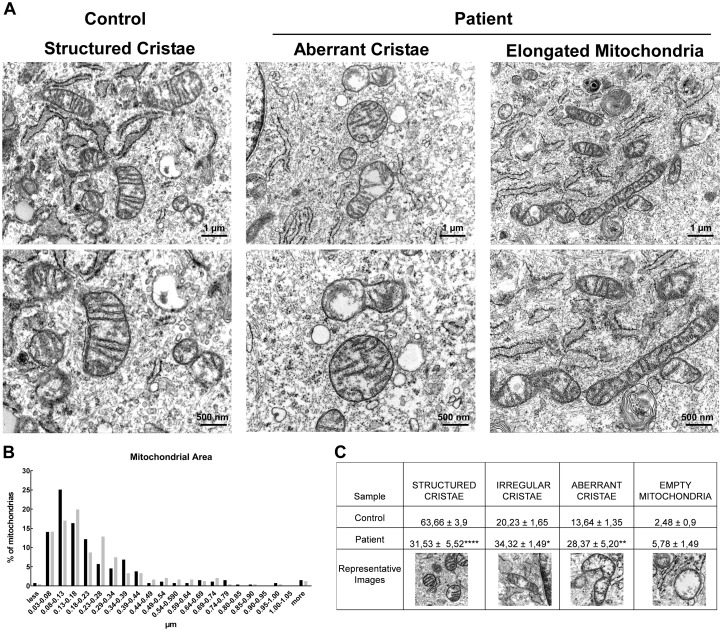
Mitochondrial ultrastructure in control and patient (p.Q92* in *MIEF2*) fibroblasts. (**A**) Cells were pelleted and fixed with glutaraldehyde 2%. The data show a population of normal mitochondria in control cells, displaying regular shape and cristae (left-hand panel). *MIEF2* p.Q92* fibroblasts show a diverse pattern, including elongated and mitochondria carrying aberrant cristae with total or partial absence of cristae (red arrows). The images are representative of 2 independent experiments. (**B**) Mitochondrial area frequency distribution, indicating an increased population of enlarged mitochondria (*n=*at least 250 mitochondria/condition). (**C**) Mitochondrial cristae were classified as: structured (showing regularly presented, deeply folded), irregular, aberrant and empty (absence of cristae).

To evaluate the ultrastructure of mitochondria from control and patient’s fibroblasts, we performed TEM studies (Fig. 5). We measured the area of mitochondria, finding no significant differences between control and MID49 p.Q92* mutant cells, yet, the distribution of mitochondrial areas frequency showed increased proportion of groups of organelles within 0.44–0.74 μm^2^, that are among the largest groups, Figure 5B. This is consistent with the morphology changes observed by confocal fluorescence microscopy. In terms of the organelle’s membrane ultrastructure, mitochondria from control cells showed structured, regular and deeply folded cristae. Strikingly, the MID49 mutant cells displayed elevated percentage of irregular cristae, consisting of short and discontinuous folding throughout a mitochondrion’s matrix with clear or empty areas, as well as a group of empty mitochondria, significantly increased, compared with control cells. Thus, the absence of MID49 in mitochondria results in an elevated population of organelles showing aberrant mitochondrial cristae organization, yet, based on our functional data, this pattern does not significantly alter overall mitochondrial function in fibroblasts.

## Discussion

One of the unique characteristics of mitochondria is their ability to build large interconnected and intracellular networks via fusion and fission (19). These two balanced activities determine the mitochondrial morphology and distribution. DRP1 oligomerizes around the mitochondrion and plays a major role in mitochondrial fission by directing this process. However, it is still unclear how DRP1 is recruited to the mitochondrial surface. To date, four different adaptors have been suggested to act as mediators of DRP1 recruitment; initially FIS-1and MFF and recently MID49 and MID51 (19). Mutations in *DRP1* lead to several serious neurological disorders including epileptic encephalopathy (20–23) and optic atrophy (24), A truncating mutation in *MFF* was first described in a Saudi Arabian child with developmental delay, pyramidal signs, and neuropathy. The mutation removes the transmembrane domain in MFF which abolishes the mitochondrial function resulting in a phenotype consistent with lack of fission (25). Finally, patients carrying MFF-loss of function variants present encephalopathy, optic atrophy and peripheral neuropathy (26).

Here, we describe a patient with a homozygous nonsense mutation in the gene encoding MID49. Although most mitochondrial fusion/fission related diseases affect the nervous system (optic atrophy, peripheral neuropathy), the phenotype of our patient is an isolated mitochondrial myopathy and does not affect optic and peripheral nerves or any other organs. The patient had a myopathy with increased CK, suggesting a metabolic myopathy, however, the muscle biopsy revealed severe mitochondrial changes with numerous ragged red fibres and COX negative fibres. Elevated CK is unusual in other forms of mitochondrial myopathies except for *TK2* deficiency and therefore it may be an important hallmark of this disease. The homozygous stop mutation (c.274C > T, p.Q92*) in *MIEF2* results in reduced protein levels of MID49 although some residual protein was still detected in immunoblot analysis in the patient’s skeletal muscle. Different antibodies targeting the MID49 protein were used in immunoblotting, however, the results were the same (data not shown). It is hypothesized that either the mutation allows some read-through, or the antibodies used for the detection of MID49 might not be strictly specific for this protein and may detect the MID51 protein due to their sequence similarity.

The defect in MID49 resulted in a muscle-specific mitochondrial dysfunction. Interestingly, in adult human skeletal muscle, *MIEF2* mRNA shows significant elevation, with regards to *MIEF1*, suggesting a relevance for this fission anchor in muscle (17). In this work, we detected reduced activities of the mitochondrial respiratory chain complexes I and IV and immunoblotting showed lower levels of multiple complexes and subunits in skeletal muscle. The several ragged red fibres and the increased mtDNA copy number and mitochondrial housekeeping protein mass indicated compensatory mitochondrial proliferation. The significantly increased levels of proteins involved in mitochondrial fusion (MFN2 and OPA1) and significantly decreased levels of DRP1 expression in skeletal muscle suggest that reduced levels of MID49 result in imbalanced mitochondrial dynamics. Significantly increased protein expression of MFN2 and OPA1 in patient muscle illustrates overexpression of proteins responsible for mitochondrial fusion at the OMM. Importantly, elevated levels of OPA1 are associated with aberrant mitochondrial morphology in cells (27), suggesting that this adaptive response in the muscle might be detrimental to mitochondrial morphology and function quality. On the other hand, DRP1 protein levels were significantly decreased in the same tissue suggesting limited mitochondrial fission activity. Although previous studies have shown that loss of MID49 in mouse embryonic fibroblast (MEF) cells does not affect the levels of MID51 (28), we show significantly increased levels of MID51 in the skeletal muscle of our MID49 deficient patient. MID49 and MID51 crystal structures show both similarities and differences. Although both MID49 and MID51 contain a nucleotidyl transferase domain, only MID51 is able to bind nucleotide diphosphates (ADP and GDP) whereas MID49 is believed to bind an unknown ligand. MID51 is dimeric in contrast to MID49 which is monomeric but both share motifs interacting with DRP1. Their structural differences might suggest a differential regulation and stabilization of MID51 versus MID49 (29–31).

Although we detected significant changes in respiratory chain function in skeletal muscle only, a milder involvement of respiratory chain function was also present in the patient’s fibroblasts as shown by the increased oxidative phosphorylation in the patient`s fibroblasts. Based on these data, the increased mtDNA copy number in fibroblasts accompanied with increased relative expression of the protein complexes of the respiratory chain and no significant mitochondrial dysfunction might indicate a possible adaptive mechanism in fibroblasts. Interestingly, we have previously demonstrated increased mtDNA levels in blood cells from Charcot Marie Tooth 2A patients, carrying *MFN2* mutations (32). In support of the dysfunctional MID49 in fibroblasts, we detected elongated mitochondria and significantly increased fusion events when compared with controls. This is in accordance with previous reports showing that the absence or downregulation of MID49 leads to and hyper-elongated mitochondrial pattern (15–16,18), however, it is the first report demonstrating that mitochondrial fusion frequency is elevated when MID49 is absent. The increased fusion events in the fibroblasts carrying the homozygous nonsense mutation were accompanied by slight increase of OPA1 and MFN2 protein levels, that might account for the elevated fusion activity. These data are consistent with previous publications where knockdown of MID49 in either COS or Hela cells resulted in increased levels of OPA1 (16). In addition, the elongation pattern displayed upon MID49 overexpression relay on the co-expression of MFN1 and MFN2 in MEF cells (18). Interestingly, the elongated mitochondrial pattern observed in MID49 or MID51 knock out cells has been linked to the recruitment of an inactive phosphorylated form of DRP1 at Ser-637 (15). Our data show no change in the overall protein levels of DRP1 in the patient’s cells, which is in agreement with previous observations (16,18). Yet, based on our data and the literature, additional mechanisms might be supporting the mitochondrial elongated phenotype, such as co-transcriptional regulation of both groups of fusion and fission proteins, or stabilization of MID51 and mitofusins or OPA1 once inserted in the corresponding mitochondrial membranes. Importantly, we demonstrate here that rescuing of MID49 protein levels recover mitochondrial morphology and dynamics, confirming that the c.274C > T, p.Q92* mutation causes the abnormal mitochondrial hyperfusion phenotype.

Finally, although the absence of MID49 did not compromise the patient’s fibroblasts oxidative performance, the ultrastructure of mitochondrial cristae showed alterations in terms of abundance and depth, displaying a significant increase of aberrant mitochondria. Conversely, studies in MID49/51 double knock-out cells showed no change in mitochondrial ultrastructure, and prevented ultrastructure re-arrangement induced by an apoptotic stimulus (33). The patient’s fibroblasts show severely reduced MID49 protein, comparable MID51 levels and only a mild increase in Opa1, in comparison to control cells, thus, the ultrastructure changes cannot be attributed to imbalanced fusion fission proteins, but presumably to the perturbation of cristae junction regulatory pathways. Also, based on the latter, the compensatory elevation of mtDNA levels stands a possible mechanism to adapt to disorganization of IMM folding, known to host OXPHOS complexes (34).

In conclusion, c.274C > T, p.Q92* in MID49 is the first reported mutation in this new adaptors of DRP1 linked to human disease. These findings emphasize the vital role of MID49 in mitochondrial dynamics, which predominantly affects mitochondrial function in skeletal muscle and displays an adaptive phenotype in fibroblasts. Our data provide new insights to the relevance of mitochondrial dynamics to cellular homeostasis.

## Materials and Methods

### Patient

The index patient was referred to a national diagnostic and clinical centre in Freiburg. Written informed consent was taken by standard procedures for total genomic DNA extraction from whole blood as well as skin and muscle biopsies.

### Muscle biopsy analysis

A muscle sample was obtained from vastus lateralis and subjected to routine histological and histochemical staining including cytochrome *c* oxidase (COX), succinate dehydrogenase (SDH), and sequential COX-SDH staining. Mitochondrial respiratory chain complex activities in skeletal muscle were measured as described previously (35).

### Molecular genetic studies

#### Mitochondrial DNA studies

Diagnostic testing for point mutations, deletions and depletion of the mtDNA were performed by standard methods. Whole exome sequencing (WES) was performed in the affected individual using Agilent SureSelect Human all Exon V4 and sequenced on an Illumina HiSeq2000 platform with 100 bp paired-end reads. Bioinfomatic prioritisation of genetic variants and segregation analysis of the likely causal mutation was performed as previously described (36). Variants identified by WES were confirmed by Sanger sequencing using custom-designed primers.

#### Quantitative real time PCR

RNA from control and patient fibroblasts was extracted using the RNeasy mini kit (Qiagen, Manchester, UK) and cDNA was generated using High-Capacity cDNA Reverse Transcription Kit (Life Technologies Ltd, Paisley, UK). Quantitative real time PCR was performed using iTaq™ universal SYBR^®^ Green supermix (primers listed below) on a CFX96 Touch™ PCR system (BioRad, Hertfordshire, UK). *MIEF1* 5′-AGG ATG ACA ATG GCA TTG GC-3′ (forward) and 5′-CCG ATC GTA CAT CCG CTT AACT-3′ (reverse); for *MIEF2*, 5′-GCA ACC AAT CCA CCA ACA GAA T-3′ (forward) and 5′-CCG GAA AAG GCG TTA AGT CAC-3′ (reverse); *β-Actin* 5′-GATGCAGAAGGAGATCACTGC-3′ (forward) and 5′-ACATCTGCTGGAAGGTGGAC-3′ (reverse); *GAPDH* 5′-CTGACTTCAACAGCGACACC-3′ (forward) and 5’-ATGAGGTCCACCACCCTGT-3’ (reverse).

#### Primary cell culture

Primary fibroblast cultures of the patient and controls were obtained from the Newcastle Biobank. All studies were carried out with informed consent of the patient and his parents and were approved by institutional ethics review boards. Fibroblasts were grown in high glucose Dulbecco’s modified Eagle’s medium (DMEM) (Life Technologies Ltd, Paisley, UK) supplemented with 10% fetal bovine serum (Sigma-Aldrich, Dorset, UK).

#### Oxygen consumption rate

Oxygen consumption rate (OCR) in fibroblasts was measured with a XF96 Extracellular Flux Analyser (Seahorse Bioscience, Agilent Technologies, Cheshire, UK), as described previously (37). Each cell line was seeded in 12 wells of a XF96-well cell culture microplate (Seahorse Bioscience) at a density of 30×10^3^ cells/well in 80 µl of DMEM and incubated for 24 h at 37°C in 5% CO_2_ atmosphere. After replacing the growth medium with 180uL of bicarbonate-free DMEM (pre-warmed at 37°C), cells were pre-incubated for 30 min before starting the assay procedure. Oxygen consumption rate (OCR), leaking respiration (LR), maximal capacity respiration (MCR) and not electron transport chain respiration (NMR) were determined by adding 1 μM oligomycin (LR), carbonyl cyanide-p-trifluoromethoxyphenylhydrazone (FCCP) (MCR: 2 injections of 0.5 μM and 1 μM, respectively) and 1μM Rotenone/antimycin (NMR), respectively (all Sigma-Aldrich, Dorset UK). The data were corrected by the NMR and expressed as pmol of oxygen/min/mg of protein. The quantity of protein was measured by Bradford assay.

#### Immunoblotting

Total protein lysates and subcellular fractions were prepared from muscle homogenates and primary fibroblasts from the index patient and two controls. Protein samples were separated on NuPAGE™ Novex™ 4–12% Bis-Tris Protein Gels. Following electrophoresis, the separated proteins were transferred onto PVDF membrane using the iBlot^®^ 2 Dry Blotting System (Thermo Fisher Scientific, UK) according to manufacturer’s instructions. The membrane was then probed for the following proteins: Total OXPHOS Rodent WB antibody cocktail (Abcam, ab110413), Anti-DRP1 (ab56788), Anti-SMCR7L (MID51) (ab89944), Anti-OPA1 (ab119685), Anti-Mitofusin 2 (ab56889), Anti-SMCR7 (MID49) (Proteintech, 16413–1-AP).

#### Analysis of mitochondrial matrix continuity and fusion event frequency

Transfection of 1–2 μg of plasmid DNA was performed by Lipofectamine 2000 (Invitrogen) following the provider’s recommendations. We used mitochondrial matrix-targeting fluorescent proteins, carrying the localization domain of cytochrome c oxidase subunit VIII: mtDsRed (Takara Bio Inc.) and mtPA-GFP (38).

In vivo imaging assays were conducted in 0.25% BSA–extracellular medium (121 mM NaCl, 5 mM NaHCO_3_, 4.7 mM KCl, 1.2 mM KH_2_PO_4_, 1.2 mM MgSO_4_, 2 mM CaCl_2_, 10 mM glucose, and 10 mM, Na-Hepes, pH 7.4, 35°C). We used a Nikon Eclipse C2 confocal microscope, located at the Advanced Microscopy Facility UC, to perform time series of cells expressing mtPA-GFP and mtDsRed, using 488-nm and 568-nm (or 561) laser lines, every 5 s. The regions of interest were illuminated by a 408-nm laser to photoconvert mtPAGFP. Continuity of mitochondria was evaluated by following the spreading of PA-GFP from the area of photoactivation, as previously described (39). Mitochondrial fusion rate was evaluated as previously described (40) and expressed as fusion events/run/min, where one run represents three ROIs. Alternatively, MID49 rescue experiments were tested by mitochondrial continuity applying FRAP assays. Regions of 5×5 μm were illuminated by 408 and 488 nm lasers in cells expressing mtDsRed. Human *MID49*-GFP cDNA construct was kindly donated by Mike Ryan, Monash University (28). The expression of the fusion protein was confirmed by immunoblotting, using anti-GFP antibody (Clontech, #632592). Image analysis was done in Fiji Image J.

#### Transmitted electron microscopy

Cultured fibroblasts were harvested within 24 after media replacement. Pellets containing 8×10^5^ cells were fixed with 2.5% glutaraldehyde and stained according to Csordas *et al*. (41). Ultrathin sections were observed at a Phillips Tecnai 12 Biotwin electron microscope, at the Microscopy Facility UC, Chile. The morphometric analysis was performed by Fiji Image J.

### Statistical analysis

Unpaired, 2-tailed *t* test was used to measure the level of statistical significance. Data are presented as ± standard error of the mean (SEM). A *P*-value of ≤0.05 was considered significant.

## Supplementary Material

Supplementary Material is available at *HMG* online.

*Conflict of Interest statement.* None declared.

## Funding 

Wellcome Trust Investigator (109915/Z/15/Z), Wellcome Centre for Mitochondrial Research (203105/Z/16/Z), Medical Research Council (UK) (MR/N025431/1), European Research Council (309548) to RH. Wellcome Trust Pathfinder Scheme (201064/Z/16/Z), Newton Fund (UK/Turkey, MR/N027302/1), European Union Seventh Framework Programme (FP7/2007-2013, 305444 RD-Connect and 305121 Neuromics) to HL. Wellcome Trust Senior Fellow in Clinical Science (101876/Z/13/Z), UK NIHR Senior Investigator, Medical Research Council Mitochondrial Biology Unit (MC_UP_1501/2), EU FP7 TIRCON, and the National Institute for Health Research (NIHR) Biomedical Research Centre based at Cambridge University Hospitals NHS Foundation Trust and the University of Cambridge to PFC. Medical Research Council (UK) Centre for Translational Muscle Disease (G0601943) to RH, HL and PFC. FONDECYT 1150677 to VE. Funding to pay the Open Access publication charges for this article was provided by the Wellcome Trust.

## Supplementary Material

Supplementary Figure 1

Supplementary Figure 2
