# Supplementary material for: A novel mechanism causing imbalance of mitochondrial fusion and fission in human myopathies
Source: Hum Mol Genet. 2018 Jan 19;27(7):1186–95. doi: 10.1093/hmg/ddy033 (PMC6159537; doi:10.1093/hmg/ddy033)
Supplement: Supplementary Figure 1 [file ddy033_supplementary_figure_1_revised.pptx]

## Slide 1
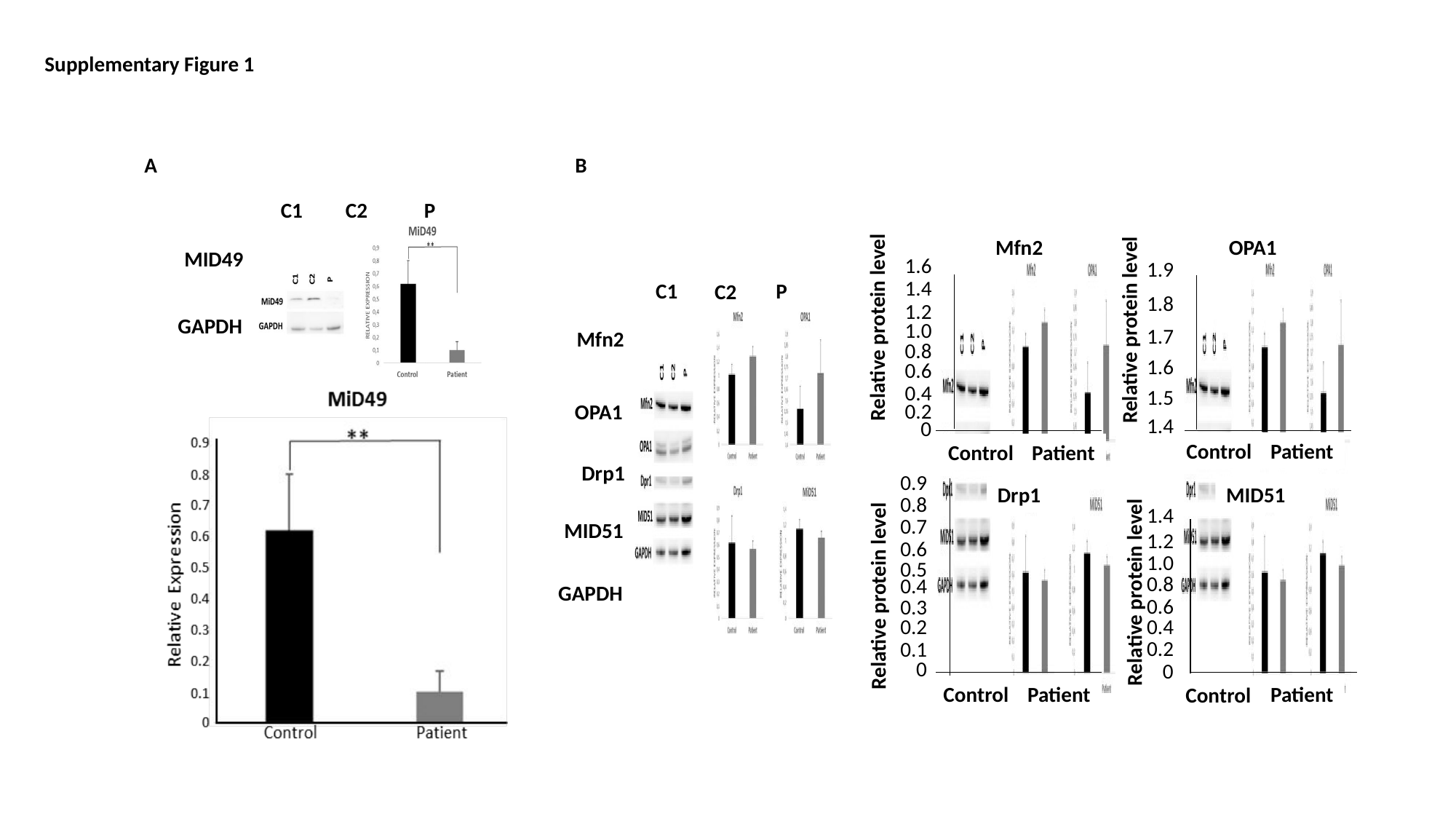

Supplementary Figure 1
A
B
C1
C2
P
MID49
GAPDH
Mfn2
OPA1
1.6
1.9
1.4
1.8
1.2
Relative protein level
Relative protein level
1.0
1.7
0.8
1.6
0.6
0.4
1.5
0.2
1.4
0
Control
Patient
Patient
Control
0.9
Drp1
MID51
0.8
1.4
0.7
1.2
0.6
1.0
0.5
0.8
0.4
Relative protein level
Relative protein level
0.6
0.3
0.4
0.2
0.2
0.1
0
0
Patient
Control
Patient
Control
C1
P
C2
Mfn2
OPA1
Drp1
MID51
GAPDH
