# Supplementary figures and images for: A novel mechanism causing imbalance of mitochondrial fusion and fission in human myopathies
Source: Hum Mol Genet. 2018 Jan 19;27(7):1186–95. doi: 10.1093/hmg/ddy033 (PMC6159537; doi:10.1093/hmg/ddy033)

## Slide 1
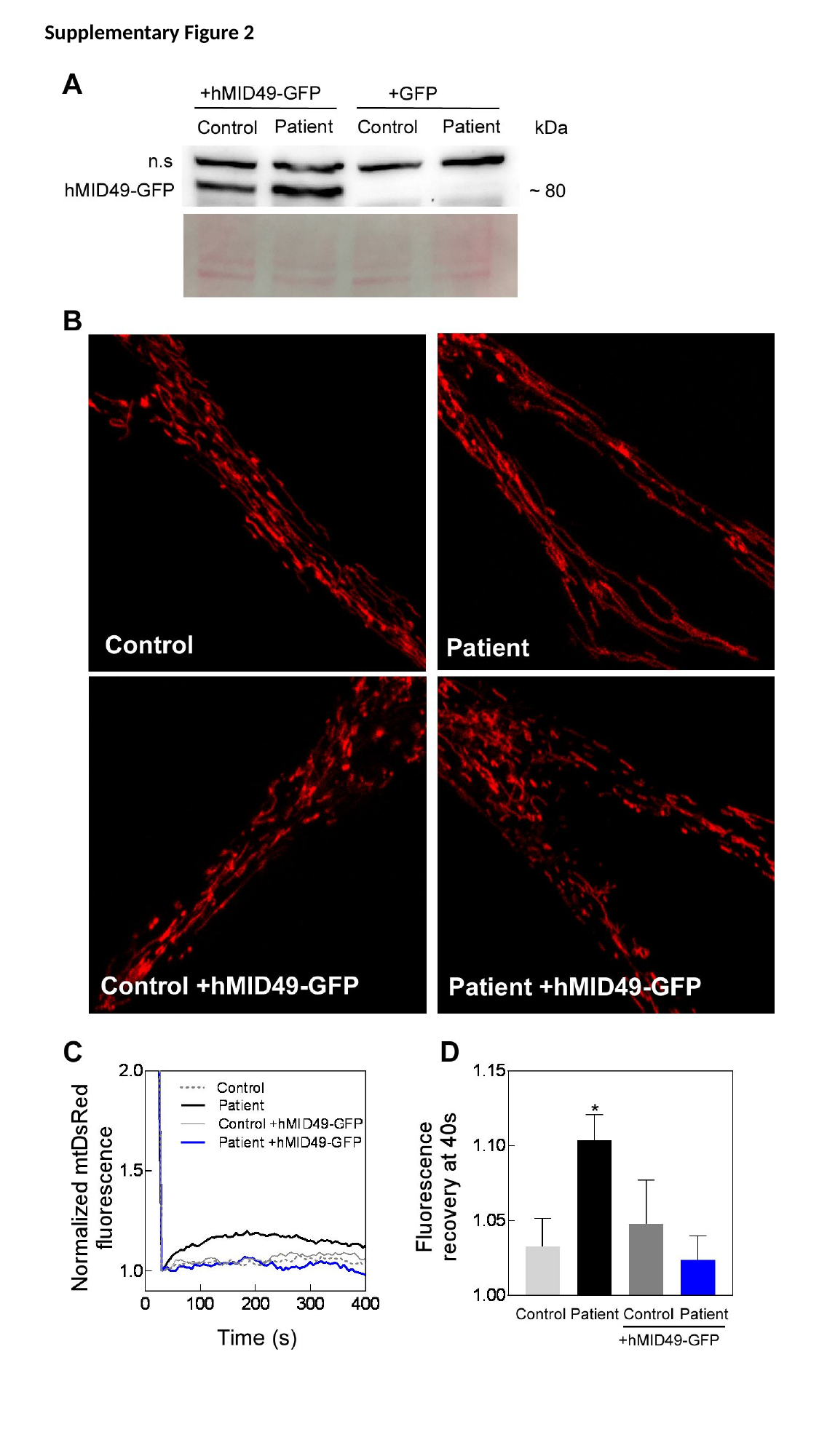

Supplementary Figure 2

Supplement: Supplementary Figure 2 [file ddy033_supplementary_figure_2_revised.pptx]
